# Antibacterial and antioxidant potentials of larval extract of black soldier fly, *Hermetia illucens* and blow fly, *Lucilia sericata*, for biomedical applications

**DOI:** 10.5114/bta/214379

**Published:** 2026-03-19

**Authors:** Shafia Mustafa Marzan, Nadim Sharif, Md. Zahidul Islam, Md. Nur Alam, Shuvra Kanti Dey, Md. Golam Mostafa

**Affiliations:** 1 Department of Biotechnology and Genetic Engineering, Jahangirnagar University, Savar, Dhaka-1242, Bangladesh; 2 Department of Microbiology, Jahangirnagar University, Savar, Dhaka-1242, Bangladesh; 3 Department of Pharmacy, Jahangirnagar University, Savar, Dhaka-1242, Bangladesh; 4 Department of Zoology, Jahangirnagar University, Savar, Dhaka-1242, Bangladesh

**Keywords:** black soldier fly, blow fly, larval extracts, antibacterial activity, antioxidant activity

## Abstract

**Background:**

This study was conducted to assess the antibacterial and antioxidant activities of the larval extracts of whole black soldier fly, *Hermetia illucens*, and green bottle blow fly, *Lucilia sericata*.

**Materials and methods:**

The disk diffusion method was used to determine the antibacterial activities of larval extracts of black soldier fly and blow fly. Five concentrations of the methanol extracts of larvae of these fly species (0, 40, 80, 160, and 325 mg/ml) were prepared in dimethyl sulfoxide and tested against Gram-positive and Gram-negative bacteria. The antioxidant activity was determined by DPPH free radical scavenging assay using four different concentrations, with ascorbic acid as the standard.

**Results:**

The disc diffusion assay showed that the larval extracts of both fly species possess strong antibacterial activity against *Bacillus subtilis, Staphylococcus aureus, Escherichia coli*, and *Salmonella enterica.* The highest antibacterial activity was observed at 80 mg/ml concentration. However, an increase in concentration beyond 80 mg/ml did not increase the antibacterial activity. Significant differences were observed between the effects of the two larval extracts, concentrations, and tested bacterial species (*p* < 0.05). The DPPH free radical scavenging assay of the larval extracts of both flies showed moderate antioxidant activities.

**Conclusions:**

The methanol extracts of larvae of two insect species exhibited strong antibacterial activities against both Gram-negative and Gram-positive bacteria and moderate antioxidant activities, indicating their pharmacological potential for use as a natural antibacterial and antioxidant source for health benefits.

## Introduction

Insects and insect-derived products are used worldwide for various purposes, such as antioxidant, antimicrobial, antidiabetic, antiobesity, antihypertensive, and antilipidemic compounds; a novel and eco-friendly food option for human consumption (Bairagi 2019; Brai et al. 2025; Alejandro Ruiz et al. 2025); and an alternative protein source for fish meal and livestock feed (Bovera et al. 2028; Cutrignelli et al. 2018; Loponte et al. 2017). Two dipteran insects – black soldier fly (BSF), *Hermetia illucens* (family *Stratiomyidae*) and the general green bottle blow fly, *Lucilia sericata* (family *Calliphoridae*) – are medically and forensically important fly species. Forensically, these flies can be used to estimate human postmortem intervals (Pujol-Luz et al. 2008). In medical science, antimicrobial peptides from *H. illucens* have been reported as a promising substitute to antibiotics in livestock farming (Xia et al. 2021). The larvae and pupae of BSF can be used as a protein substitute source for livestock feed, aquaculture, human nutrition, and pet food (Kuppusamy et al. 2020; Rumpold et al. 2013; Raheem et al. 2018) because the protein and lipid contents in BSF larvae are 40–50% and 35%, respectively (Makkar et al. 2024; Wang and Shelomi 2017). The blow fly larvae are also popular for application in veterinary, forensic, medical, and agricultural fields (Blenkiron et al. 2015; Khater et al. 2011; Lord et al. 1994; Mostafa et al. 2020; Shin et al. 2021; Tarone et al. 2011). These larvae contain approximately 51% crude protein and 20% fat and are a good source of animal feed (Church 1996). The blow fly larvae, also known as wound maggots or medical maggots, are used to cure wounds in traditional medicine (Church 1996; Pöppel et al. 2015). The larvae of both BSF and blow fly produce antibacterial peptides (Pöppel et al. 2015; Kim et al. 2020; Parry et al. 2021), which can bind to the cell wall of various microbial pathogens, including Gram-negative and Gram-positive bacteria (Choi et al. 2012; Park et al. 2014b). Blow fly is broadly used for maggot therapy of wounds with sterile maggots, while their larval excretions and secretions are utilized for clearing necrotic tissues and preventing bacterial proliferation in wounds (Davies et al. 2013).

Blow fly maggots have been effectively used as a debridement agent for chronic and infected wounds from ancient times (Abdel-Reheem 2016). Previous studies have demonstrated that debridement therapy using larvae of blow fly maggots can cure chronic ulcers, venous leg ulcers, foot ulcers, and diabetes. These larvae have also been utilized as waste management and bio-recycling organisms (Wang and Shelomi 2017), which convert organic wastes, pollutants, and substrates of food or animal wastes into proteins and fats (Pöppel et al. 2015; Kim et al. 2020; Parry et al. 2021; Erickson et al. 2004; Lalander et al. 2013).

They also serve as alternative protein and fat sources of soymeal and fishmeal and are considered safe and cost-effective human food and livestock feed (Kim et al. 2020; Hale 1973; Shumo et al. 2019). Approximately 2000 insect species are consumed in around 113 countries worldwide (Yen 2010). Among these species, BSF larvae have food processing properties, including fat binding capacity and protein solubility (Bubler et al. 2016).

The antibacterial properties of insects have attracted their use in nutritional immunology (Park et al. 2014b), and the potential antibacterial proteins from these insects could be regarded as natural animal and human food additives and bio-preservatives (Solomons 1978). However, in every major livestock-producing country, animal feeds are widely supplemented with antibiotics at subtherapeutic concentrations to increase the rate of animal development and feed productivity, disease therapy, and disease prophylaxis (Martin et al. 2014). Moreover, despite having nutritional advantage, animal feeds can pose potential dangers because of accumulation of heavy metals and toxins in their sources (Blenkiron et al. 2015).

Due to concerns related to human and animal health hazards and emergence of antibiotic resistance or low efficacy (Martin et al. 2014), the use of antibiotics in animal feed and animal husbandry has been restricted in many European countries (Millet and Maertens 2011). Therefore, finding an effective and cheap alternative to antibiotics is currently a critical issue (Rabani et al. 2019).

Oxidative stress is considered the primary factor underlying many progressive diseases, such as gastric ulcer, hyperlipidemia, cancer, and diabetes (Kumaran and Karunakaran 2007). Antioxidants, functioning as micronutrients, have recently gained increasing importance because of their capability to deactivate free radicals and thus defend the body from injury caused by free radical-induced oxidative stress (Sherman et al. 2000). Recent studies suggest that BSF and blow fly larval extracts possess antioxidant activities, and their fractions can be used to develop safe and functional foodstuff, therapeutics, and pharmaceuticals (Park et al. 2014a; Solomons 1978; Sherman et al. 2000).

Therefore, considering the forensic, pharmaceutical, therapeutic, bio-recycling, and medicinal importance as well as the potentiality of safe, nutritious, and cost-effective livestock feed, it is crucial to investigate the beneficial properties of larvae of these two fly species. The current study aimed to reveal the antibacterial and antioxidant potential of the larval extracts of BSF and blow fly.

## Materials and methods

### Collection and preparation of larvae samples

Adult individuals of BSF and blow fly were collected from Jahangirnagar University campus, Savar, Dhaka-342, Bangladesh. The blowfly species was morphologically identified as *L. sericata* based on the following taxonomic characteristics: presence of 2–5 setae on the central occipital area below the inner vertical setae; typically bright green abdomen, occasionally shining coppery; and humeral callus with 6–8 small setulae along the posterior margin (Whitworth 2006). Although most individuals tend to be green, some are coppery; moreover, they also have a setose metasternum, which is often hidden and difficult to observe (Whitworth 2019). Adult individuals of BSF have two wings and are black in color; however, they do not possess any stinger (Sheppard et al. 2002). The flies were reared in cages in the laboratory under typical temperature (29 ± 1°C) and relative humidity (80 ± 10%) and protected with an external mosquito net to prevent the entry of other insect species.

**Figure 1:**
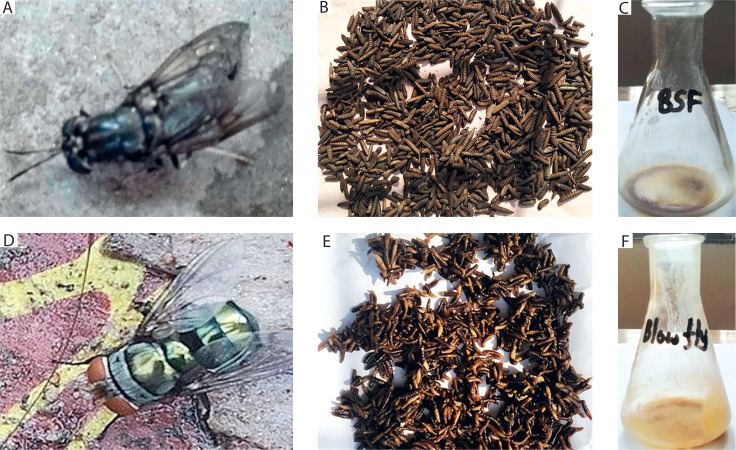
Photographs of black soldier fly (**A, B**, and **C**) and blow fly (**D, E**, and **F**). (**A, D**) Adult, (**B, E**) larvae, (**C, F**) larval extract

The larvae of both fly species were reared on kitchen garbage, which was collected from the household and canteens of the university campus. The organic waste, mainly composed of vegetables, fish, and chicken from the same source, was supplied to the larvae; the feeding rate of kitchen waste to larvae was 200–220 mg/larvae/day. The larvae were fed daily. Seven-day-old blow fly larvae and 20-day-old BSF larvae were harvested and washed in tap water followed by distilled water in a clean sieve (Figure 1). The larvae were then anesthetized by keeping them on ice for 5 min and air-dried. Next, they were oven-dried for 24 h at 65°C. The larvae of both species, weighing 50 gm, were crushed separately in a mechanical mini oil press machine (DL-ZYJ05, China) equipped with a heating jacket and an adjustable nozzle. The machine delivered crushed larvae in two forms: larvae paste and larvae cake (Rozali et al. 2022). In this technique, the whole bodies of the larvae were press squeezed in the machine; one portion of the whole body was converted into a paste containing press liquid, resulting in a larval paste; the other portion was transformed into a dry and hard larval cake. In this study, the soft larval paste was used to prepare the larval extract for further experiments.

### Methanol extracts

Briefly, 10 g larval paste of each fly species was dissolved separately in 100 ml methanol as solvent. The extracts were sieved by a Whatman filter paper, and the filtered extracts were stored at 4°C until further use. The solvent of the extracts was removed using a rotary evaporator (Hei-VAP, Heidolph, Germany).

### Antibacterial assay of the larval extracts

Bacterial culture media and the test concentrations of the larval extracts were prepared according to the method described by Auza et al. (2020) with slight modifications. Briefly, the test bacterial species were subcultured in nutrient broth medium. A total of 100 µl inoculum of bacterial culture containing approximately 1.5 × 10^8^ CFU/ml standardized against 0.5 MacFarland standard was swabbed onto Muller Hilton Agar medium (HIMEDIA, India). Simultaneously, the larval extract of BSF and blow fly were dissolved in dimethyl sulfoxide (DMSO) solution to prepare test concentrations (40, 80, 160, and 325 mg/ml), with four repetitions of each concentration. Ciprofloxacin (5 µg/disc) was used as a positive control.

The antibacterial activities of the four different test concentrations of the larval extracts were determined by the disc diffusion method (Hudzicki 2009) (zone of growth inhibition) against the following two Gram-positive and two Gram-negative bacterial strains: *Escherichia coli* ATCC 25922 and *Salmonella enterica* ATCC 35664 as Gram-negative strains, and *Bacillus subtilis* ATCC 6051 and *Staphylococcus aureus* ATCC 23235 as Gram-positive strains. Filter paper discs (5 mm diameter) were independently impregnated with 5 µg/ml of the larval extracts (each group) and placed onto the solidified agar plates previously inoculated with the test strains. The plates were incubated for 24 h at 37°C. The antibacterial activities were determined from the zone of inhibition according to the guidelines of the Clinical and Laboratory Standards Institute.

### DPPH scavenging activity

To assess the antioxidant potential of the larval extracts by the DPPH free radical scavenging assay, variations in the optical density of DPPH radicals were measured. The reagent solution of DPPH (0.5 mM) was prepared by dissolving 4 mg of DPPH in 100 ml methanol. Four experimental concentrations (200, 100, 50, and 25 µg/ml) of the samples and ascorbic acid were prepared. Next, 0.2 ml of ascorbic acid (0.1 mg/ml) and each of the sample concentrations were reacted with 2 ml of DPPH solution (0.5 mM). After 30 min of incubation, the absorbance was measured at 517 nm by using a UV-Visible double beam spectrophotometer (UV-1601(PC) 220 V, Shimadzu Corporation, Japan). The percentage of DPPH radical scavenging was calculated using the following equation:


$$\%\text{ inhibition of DPPH radical}=(\left[{\text{A}}_{0}-{\text{A}}_{1}\right]/{\text{A}}_{0})\times 100$$

where A_0_ is the absorbance of the control and A_1_ is the absorbance of the extract/standard.

### Statistical analysis

Statistical analyses were conducted with 4 replicates of each larval extract. The results are expressed as mean ± standard error (SE). The inhibitory effects of the different concentrations of the two larval extracts on the four bacterial strains were analyzed by ANOVA followed by DMRT by using SPSS software. A *p*-value of <0.05 was considered statistically significant.

## Results

### Antibacterial activity of the BSF larval extract

**Figure 2:**
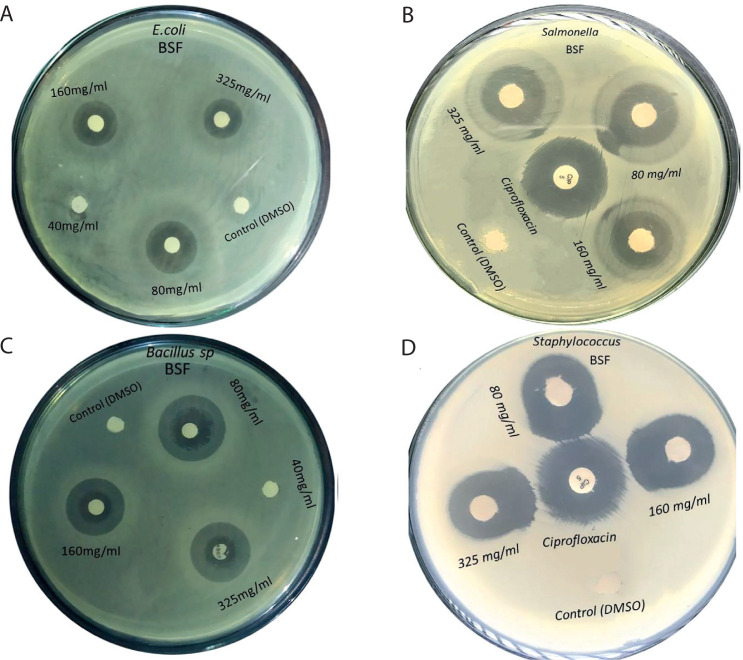
Antibacterial activities of black soldier fly larval extracts against Gram-negative and Gram-positive bacteria. (**A**) Escherichia coli, (**B**) Salmonella enterica, (**C**) Bacillus subtilis, (**D**). Staphylococcus aureus

Among the tested concentrations of the BSF larval extracts (0, 40, 80, 160, and 325 mg/ml), the 80 mg/ml concentration showed the highest mean zone of growth inhibition against *S. aureus*(23.5 mm), followed by that against *B. subtilis* (22.5 mm), *S. enterica* (16.25 mm), and *E. coli* (14.75 mm) (Table 1 and Figure 2). Although the control and 40 mg/ml concentration showed no inhibitory activity against both Gram-positive and Gram-negative bacteria, the 160 and 325 mg/ml extract concentrations showed gradually lower activities for all the tested strains.

**Table 1:** Antibacterial activities of four different concentrations of extracts of black soldier fly (BSF) against Gram-positive (*Bacillus subtilis* ATCC 6051 and *Staphylococcus aureus* ATCC 23235) and Gram-negative (*Escherichia coli* ATCC 25922 and *Salmonella enterica* ATCC 35664) bacteria

BSF [µg/disc]	*Escherichia coli*, mean ± SE	*p*-value	*Salmonella enterica*, mean ± SE	*p*-value	*Bacillis subtilis*, mean ± SE	*p*-value	*Staphylococcus aureus*, mean ± SE	*p*-value
Control*	0	4.25E^–16^	0	7.77E^–16^	0	4.31E^–15^	0	7.66E^–15^
40	0		0		0		0	
80	14.75 ± 0.47^a^		16.25 ± 0.4		22.5 ± 0.64^a^		23.5 ± 0.45	
160	13.5 ± 0.288^b^		15.25 ± 0.2		17.75 ± 0.62^b^		19.5 ± 0.31	
325	11 ± 0.40^c^		14.25 ± 0.35		16.25 ± 0.75^b^		19.5 ± 0.5	

* Ciprofloxacin (5 µg/disc)

Data represents as mean ± standard error (SE) of four replications.

Means in a column followed by the same letter do not differ significantly at 5% level of significance (DMRT).

### Antibacterial activity of the blow fly larval extract

**Figure 3:**
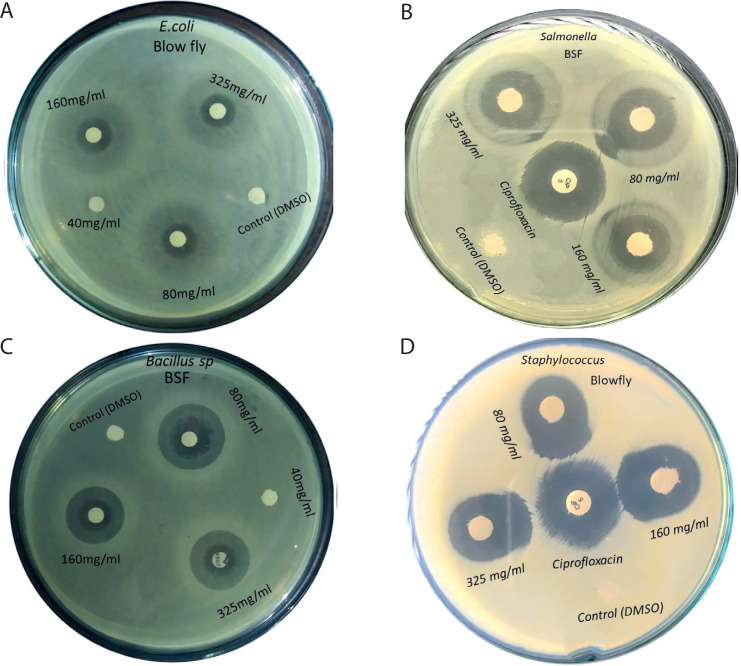
Antibacterial activities of green bottle blow fly larval extracts against Gram-negative and Gram-positive bacteria. (**A**) Escherichia coli, (**B**) Salmonella enterica, (**C**) Bacillus subtilis, (**D**). Staphylococcus aureus

Among the abovementioned tested concentrations of the blow fly larval extracts, the 80 mg/mL concentration showed the highest mean zone of growth inhibition (20.75 mm) against *S. aureus*, followed by that against *B. subtilis* (18.75 mm), *S. enterica* (14.75 mm), and *E. coli* (11.0 mm) (Table 2 and Figure 3).

**Table 2:** Antibacterial activities of four different concentrations of extracts of green-bottle blow fly against (*Bacillus subtilis* ATCC 6051 and *Staphylococcus aureus* ATCC 23235) and Gram-negative (*Escherichia coli* ATCC 25922 and *Salmonella enterica* ATCC 35664) bacteria

Green-bottle blow fly [µg/disc]	*Escherichia coli*, mean ± SE	*p*-value	*Salmonella enterica*, mean ± SE	*p*-value	*Bacillis subtilis*, mean ± SE	*p*-value	*Staphylococcus aureus*, mean ± SE	*p*-value
Control	0	7.86E^–14^	0	1.78E^–13^	0	6.22E^–15^	0	8.27E^–12^
40	0		0		0		0	
80	11.00 ± 0.40^a^		14.75 ± 0.40		18.75 ± 0.47^a^		20.75 ± 0.45	
160	10.00 ± 0.40^ab^		13.75 ± 0.20		16.50 ± 0.64^b^		18.50 ± 0.31	
325	9.25 ± 0.47^b^		13.25 ± 0.35		16.00 ± 0.70^b^		19.00 ± 0.50	

* Ciprofloxacin (5 µg/disc).

Data represent as mean ± standard error (SE) of four replications.

Means in a column followed by the same letter do not differ significantly (p > 0.05, Duncan’s multiple range test).

Although the control and 40 mg/ml concentration showed no inhibitory activity against both Gram-positive and Gram-negative bacteria, the 160 and 325 mg/ml concentrations exhibited gradually lower activities.

### Comparison of the antibacterial activities of BSF and blow fly larval extracts

The BSF larval extract exhibited higher antibacterial activity against both Gram-positive and Gram-negative test strains compared to the blow fly larval extract. However, both larval extracts showed strong antibacterial activities against Gram-positive bacteria than against Gram-negative bacteria.

Furthermore, the antibacterial activity of the larval extracts of BSF and blow fly increased with increasing concentrations. For all four test strains, the antibacterial activity was first observed at 80 mg/ml concentration but gradually decreased with the increase in concentration from 160 to 325 mg/ml. The larval extract concentrations displayed varying antimicrobial activity. The reason for this variation might be differences in interaction between the bacterial nucleus and cell wall components and the active compounds present in the larval extracts (Choi et al. 2012). Although BSF is a non-vector and non-pathogenic fly species, is eco-friendly and nontoxic to health, and does not carry disease-causing pathogens, blow fly species serves as a vector to pathogens.

According to ANOVA analysis, for both BSF and blow fly larval extracts, the 80 and 160 mg/ml extract concentrations showed significant differences in the inhibition zone diameter for Gram-positive bacteria *B. subtilis* and *S. aureus* and Gram-negative bacteria *S. enterica* and *E. coli* (*p* < 0.05). This result implies that the different extract concentrations showed differences in inhibiting the growth of the test bacterial strains. The BSF larval extract concentrations showed significant differences in inhibiting the growth of *E. coli* (*p* = 4.25E^–16^), *S. enterica* (*p* = 7.77E^–16^), *S. aureus* (*p* = 7.66E^–15^), and *B. subtilis* (*p* = 4.31E^–15^). The blow fly larval extract concentrations also showed significant differences in inhibiting *E. coli* (*p* = 7.86E^–14^), *S. enterica* (*p* = 1.78E^–13^), *S. aureus* (*p* = 8.27E^–12^), and *B. subtilis* (*p* = 6.22E^–15^). Thus, the present study confirmed that the larval extracts of both fly species have potential to inhibit bacterial growth.

### Minimum inhibitory concentration (MIC) of the larval extract

The MIC of the BSF larval extract against *S. enterica, S. aureus, E. coli,* and *B. subtilis* was 60 mg/ml, while that of blow fly larval extract was 80 mg/ml. These MIC values provide insights into the dose required to effectively inhibit bacterial growth, helping to avoid under- or over-dosing.

### Antioxidant activity of blow fly and BSF larval extracts

**Figure 4:**
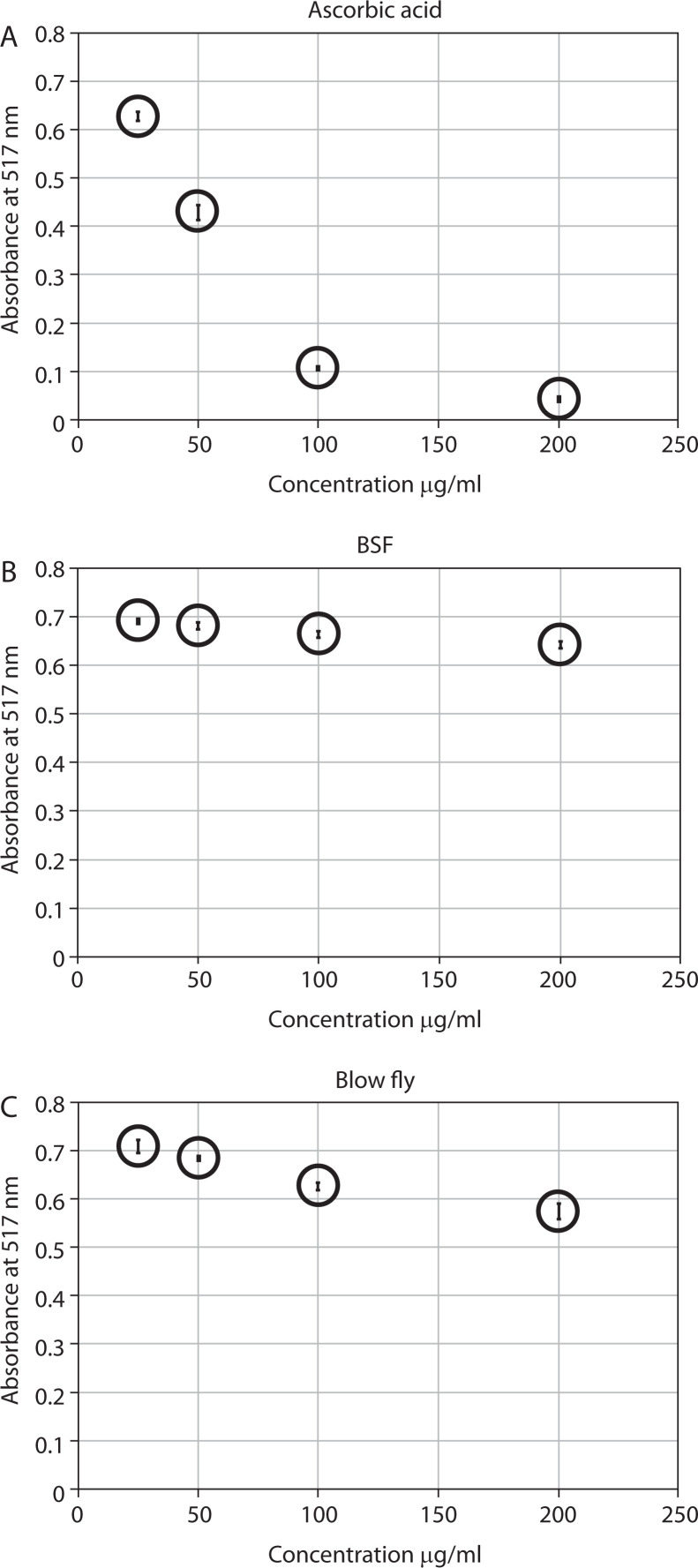
(**A**) DPPH radical scavenging activity of various concentrations of ascorbic acid. (**B**) DPPH radical scavenging activity of various concentrations of BSF larval extract. (**C**) DPPH radical scavenging activity of various concentrations of blow fly larval extract

The DPPH free radical scavenging assay showed that the blow fly larval extract exhibited poor antioxidant activity compared to standard ascorbic acid. The reduction capability of DPPH radicals was measured by the decrease in its absorbance at 517 nm induced by the antioxidants from the extract and ascorbic acid (Figure 4). A dose-dependent increase in the percentage of antioxidant activity was noted for all tested concentrations. The blow fly larvae extract at four tested concentrations (200, 100, 50, and 25 µg/ml) exhibited 24.06%, 19.30%, 11.45%, and 8.62% inhibition of DPPH radicals, respectively. In contrast, the BSF larval extract at the same tested concentrations displayed 17.50%, 14.41%, 11.96%, and 10.29% inhibition of DPPH radicals, respectively (Table 3). Thus, the blow fly larval extract showed comparatively higher antioxidant activity than the BSF larval extract. Although the IC_50_ values of both larval extracts could not be achieved, they showed mild antioxidant activities.

**Table 3:** Percentage inhibition of DPPH as free radical scavenging activity at different concentrations of methanolic extract of black soldier fly, green-bottle blow fly and standard (ascorbic acid)

Concentration [µg/ml]	% Inhibition of DPPH by black soldier fly larvae extract	% Inhibition of DPPH by green-bottle blow fly larvae extract	% Inhibition of DPPH by standard
200	17.5	24.0	92.3
100	14.4	19.3	87.5
50	11.9	11.4	48.8
25	10.3	8.6	20.0

## Discussion

Microbial infections are severely hazardous for humans and animals (Krupodorov et al. 2022). Although antibacterial drugs have been traditionally used to control microbial infections (Tanhaeian et al. 2020), their effectiveness has become a cause of concern in recent years due to the emergence of antibacterial resistance and multidrug-resistant bacteria (Dunai et al. 2019; Fair and Tor 2014) as well as the side effects of drugs, including allergic reaction, nausea, vomiting, diarrhea, bloating, indigestion, and abdominal pain (Martin et al. 2014). To ensure safe and nutritious food for humans and livestock feed and find an alternative to antibiotics, the present study explored the antibacterial and antioxidant activities of larval extracts of two dipteran fly species. In the agar-based disc diffusion assay, five concentrations of BSF and blow fly larval extracts were tested against four standard Gram-positive and Gram-negative bacterial strains. The larval extracts inhibited the growth of both Gram-positive and Gram-negative strains. The BSF larval extract strongly inhibited the growth and multiplication of bacteria. As reported previously, the larval extract of these flies showed antibacterial activity against Gram-positive species (*S. aureus* and *Streptococcus* sp.) and Gram-negative species (*Proteus* sp., *E. coli*, and *S. typhi*) (Park et al. 2014a). In the present study, we noted that this larval extract showed optimum antibacterial activity at 80 mg/ml concentration. Further in-depth studies are required to understand the reasons for microbial growth inhibition at this optimum concentration.

In a previous study, methanolic extracts of BSF demonstrated inhibitory activity against *S. typhimurium, E. coli*, and *P. aeruginosa* at 75, 125, 175, 225, 275, and 325 mg/ml concentrations (Auza et al. 2020). In a similar study on the potential antibacterial activities of *H. illucens* oils, *B. subtilis* and *S. aureus* were selected as representative Gram-positive bacteria, and *E. coli* and *P. aeruginosa* were chosen as Gram-negative bacteria. Both Gram-negative species showed complete resistance, while the Gram-positive strains displayed little sensitivity (Blenkiron et al. 2015; Fair and Tor 2014).

Based on the inhibition zone diameter, the inhibitory activity is classified as weak (5 mm), moderate (5–10 mm), strong (10–20 mm), and very strong (20–30 mm) (Detha et al. 2018; Morales et al. 2003). Considering these benchmark standards, the present study demonstrated that both larval extracts had stronger activities against Gram-positive strains than against Gram-negative strains. However, some investigators reported that the methanolic extracts of both BSF and blow fly larvae showed higher inhibition of Gram-negative bacteria than of Gram-positive bacteria. The fly larvae possess different biological characteristics and thus produce various types of compounds that exhibit inhibitory properties against different bacterial pathogens (Park et al. 2014b). The variations in the inhibitory effects of larval extracts on different bacterial species might also be attributed to differences in interaction between the bioactive compounds present in the extract and the bacterial nucleus and cell wall constituents (Choi et al. 2012; Auza et al. 2020).

In the DPPH assay, the scavenging effect of the extracts and ascorbic acid increased with the increase in concentrations. DPPH radicals are scavenged in the presence of a proton-donating substance (H^+^), leading to a change in color from purple to yellow and a reduction in absorbance value. In the current study, the larval extracts of BSF and blow fly moderately scavenged DPPH radicals, with the blow fly larval extract showing a better effect than the BSF larval extract. A slight change in purple color was observed following the reduction of DPPH radicals by the extracts; this was due to the acceptance of a hydrogen or an electron by the DPPH radical to become a stable molecule (Liu et al. 2010) expressing the antiradical activity of its substance to scavenge the DPPH radicals (Désiré et al. 2016). In a previous study, the DPPH radical scavenging assay showed that, although chicken meal containing BSF exhibited pro-oxidant activities at all tested concentrations, fishmeal with BSF displayed pro-oxidant activity at four of five tested concentrations (Mouithys-Mickalad et al. 2020). In this previous study, the IC_50_ values for the samples were not calculated either due to their pro-oxidant activity or because 50% inhibition was not achieved during the assay (Mouithys-Mickalad et al. 2020). A comparison of the DPPH radical scavenging activity of ethanol, methanol, and water extracts of BSF larvae and pupae revealed that the BSF pupae water extract displayed the highest DPPH radical scavenging activity among the samples (Park et al. 2014a). The variations in DPPH radical scavenging activities were due to differences in solvents, incubation periods during assays, insect growth stages, and insect types. Mehmood and Murtaza (2018) reported that compounds with antimicrobial activity can be extracted in both ethanol and methanol as solvents. They found that methanolic extracts exhibited higher antimicrobial potential because of the high polarity of methanol, which facilitated the extraction of all phenolic compounds (Borges et al. 2020). Jafri et al. (2023) also reported that, compared to ethanolic and water extracts, methanolic extract showed higher antioxidant and antimicrobial activity. Therefore, in the present study, methanol was used as the most suitable solvent.

Previous studies also showed that antioxidant and antimicrobial activities vary among extracts from different types of insects. The differences in antioxidant effects of insects and invertebrates could be attributed to differences in extraction solvents as well as variations in their taxonomy and dietary habits (Alejandro Ruiz et al. 2025). The drying technique of larvae was shown to be a relevant factor influencing extraction eficiency, in terms of the quantity and diversity of antioxidant compounds extracted by polar and nonpolar solvents. Nearly all solvents delivered higher extraction yields when freeze-dried larvae was used as the matrix (Keil et al. 2022). Moreover, many antioxidants that react rapidly with peroxyl radicals may react gradually with DPPH or may be inert to DPPH (Keil et al. 2022).

In the present study, we found that the larval extracts of BSF and blow fly showed antibacterial activities against two Gram-positive and two Gram-negative bacterial species; this is one of the first studies in Bangladesh that can contribute to the discovery of new antibacterial agents. The larval extracts of these flies also displayed antioxidant activities. The antibacterial and antioxidant properties of the larval extracts of BSF and blow fly observed in this study were consistent with the findings reported in previous studies. The search for new antibacterial agents against drug-resistant pathogens is one of the major challenges in treating infectious diseases of humans and animals. In this regard, the larval extracts of insects, particularly those of dipteran fly species, show promising potential to function as novel antimicrobial agents.

## Conclusions

The larval extracts of both BSF and blow fly showed potential for antibacterial and antioxidant effects, which can be further enhanced through biochemical interventions. The findings of this study provide further evidence that the larval extracts of these two insect flies have pharmacological potential for application as a natural antioxidative and antibacterial source for health benefit. However, to ensure their proper application, it is essential to identify and characterize the bioactive antibacterial and antioxidant molecules present in these larval extracts. Therefore, further research is required to incorporate this novel source in pharmacological drug design and use it as an economical and safe alternative for human consumption and livestock feed.
